# Wear Resistant Nanocomposites Based on Biomedical Grade UHMWPE Paraffin Oil and Carbon Nano-Filler: Preliminary Biocompatibility and Antibacterial Activity Investigation

**DOI:** 10.3390/polym12040978

**Published:** 2020-04-22

**Authors:** Michelina Catauro, Cristina Scolaro, Giovanni Dal Poggetto, Severina Pacifico, Annamaria Visco

**Affiliations:** 1 Department of Engineering, University of Campania “Luigi Vanvitelli”, Via Roma 29, I-81031 Aversa, Italy; 2 Department of Engineering, University of Messina, C.da Di Dio, 98166 Messina, Italy; cscolaro@unime.it; 3 Ecoricerche, Srl, Via Principi Normanni, 81043 Capua (CE), Italy; giogiodp@hotmail.it; 4 Department of Environmental, Biological and Pharmaceutical Sciences and Technologies; University of Campania “Luigi Vanvitelli”, Via Vivaldi 43, 81100 Caserta, Italy; severina.pacifico@unicampania.it; 5 Istituto per i Polimeri, Compositi e Biomateriali - CNR IPCB, Via Paolo Gaifami 18, 9-95126 Catania, Italy

**Keywords:** UHMWPE, nanocomposites, wear tests, FTIR, bio-activity

## Abstract

In the present paper, we investigate the effectiveness of nanocomposites (composed of ultra-high molecular weight polyethylene (UHMWPE) mixed with carbon nano-filler (CNF) and medical grade paraffin oil (PO), from the biological point of view. Wear measurements were carried out without (air) and with lubricant (distilled water, natural, and artificial lubricant), and antibacterial activity and cytotoxicity were evaluated. The results highlighted that the presence of CNF is important in the nanocomposite formulation because it reduces the wear rate and prevents oxidative degradation during its processing. An amount of 1.0 wt % of CNF is best because it reaches the optimal distribution within the polymeric matrix, resulting in the best wear resistant, bio-active, and anti-bacterial nanocomposite among all investigated samples.

## 1. Introduction

Biomedical grade ultra high molecular weight polyethylene (UHMWPE) has high wear/abrasion resistance toughness and biocompatibility features and has been the standard material for joint replacement (JR) in artificial knees and hips prosthesis for more than half a century to date [1].

However, UHMWPE is considered the weakest part of the artificial joint: during its use, plastic debris is produced from the wear mechanism. Debris gives rise to adverse reactions, which lead to osteolysis and aseptic loosening of the prosthesis causing the failure of the entire joint, and hence the revision surgery [2]. After implantation in the human body, indeed, UHMWPE undergoes degrading mechanisms in the physiological environment and for the sliding friction against the other metallic or ceramic harder components of the artificial mobile prostheses [3,4,5]. For this reason, the research of the last years is highly focused on the improvement of mechanical resistance of this material during its use in vitro [6,7,8,9]. Highly cross-linked ultra-high-molecular-weight polyethylene was observed to be the most clinically promising in total joint arthroplasty owing to its high wear resistance [10].

Cross-linking of UHMWPE can be initiated by free radicals, which can be introduced by various methods such as exposure to ionizing radiation (i.e., gamma rays, electron beam) or the incorporation of chemical cross-linking agents such as peroxides or silanes [11,12,13,14]. Then, an anti-oxidation treatment is required to avoid material oxidation: thermal quenching, thermal annealing, or blending with antioxidant molecules in an opportune amount [15,16]. Vitamin E has been considered as an important antioxidant for UHMWPE, preventing its oxidative degradation, and increasing its wear resistance and fatigue [17]. Anyway, if on the one hand, vitamin E has been effective as a free radical scavenger, on the other hand, it could reduce the cross-linking efficiency [18]. Recently, Oral et al. 2019 combined the consolidation and cross-linking of UHMWPE in one step, such an opportunity to manufacture highly wear and oxidation-resistant joint implant-bearing surfaces with much improved toughness. The presence of Vitamin E in the UHMWPE formulation is important for its oxidative stabilization [19].

With the aim to enhance the mechanical properties of UHMWPE, many other alternative methods have been used, such as the mixing with reinforcements (particles or fibres) [20]. It is known that graphite acts as a lubricant to increase the wear resistance in friction components. For this reason, UHMWPE reinforced with carbon nanofibers (codified as CFR-UHMWPE, or “Poly II”) has been used in orthopedic implants for total hip or total knee arthroplasty (THA/TKA), in 1970 [21]. However, this composite has been withdrawn because of its decreasing in crack resistance, of the fibers pull off on the surface, and other applicative problems. However, these nanocomposites are reconsidered in these last years, owing to the great innovations in the incorporation methods developed in the new experimentation, and also because of the cytocompatibility of the carbon nanofiller [22]. As an example, Puertolas et al. [23] evaluated the influence of carbon nanotubes (CNTs) and graphene as reinforcement fillers in UHMWPE matrix, while Yousef et al. [4] highlighted that carbon nanofiller can lead to an improvement in the wear behavior of biomedical grade polyethylene.

In our previous papers [4,24], we have shown the wear resistance features of nanocomposites made by UHMWPE mixed with 1.0 wt % of carbon nano-filler (CNF) and 2.0 wt % of medical grade paraffin oil (PO). In particular, the carbon nanofiller acts as lubricant while the paraffin oil reduces the typically high viscosity of UHMWPE, favoring its process ability and the filler dispersion [4]. These nanocomposites are innovative because they exhibited an appreciable wear resistance compared with that of graphene-filled nanocomposite, with the advantage of low cost [24].

In the present paper, we investigate the effectiveness of these nanocomposites (which had a wear behavior enhancement) from the biological point of view (antibacterial activity and cytotoxicity). Biocompatibility or cytotoxicity aspects need to be evaluated to determine if adverse reactions could have occurred as the body’s response.

In the nanocomposites, UHMWPE is mixed with PO and CNF. UHMWPE is commonly classified as bio-inert material. PO is a mineral oil and is compatible with the human body; for this reason, it is used in medical applications as well as in cosmetics [25]. Carbon filler instead needs special attention because its particles could migrate, being dangerous for the human body. In particular, if carbon filler contains dangling carbon bonds on their surface, they are considered highly reactive. Similarly, if carbon filler contains traces of residual catalysts, these could be dangerous and reactive as well. Consequently, the high wear resistance of an UHMWPE based nano-composites containing carbon fillers is the key-factor to limit the migration of the filler. In fact, the more resistant to wear the material, the lower the carbon filler load that could migrate outside in the surrounding areas of the nanocomposite. An improvement of wear resistance is very important to minimize the danger of adverse effects on implants. For these reasons, and because to date there are no studies on this aspect of these nanocomposites related to their biological features, we present the wear and biological (bioactivity and anti-bacterial) features of these materials.

## 2. Materials and Methods

The control sample (code UP) was made of pure medical grade UHMWPE (code GUR1020 Ticona^®^, Sulzbach, Germany, molecular weight = 2–4 × 10^6^ g/mol and density = 0.93 g/cm^3^) mixed with 2.0 weight% of pharmaceutical grade Paraffin oil (Sella Pharmaceutical and Chemical Laboratory, Schio (Vi), Italy), added to UHMWPE to favor its mixing and processing.

Nanocomposites were obtained by mixing pure UHMWPE (code: U) with paraffin oil (2.0 weight%—code: P) and with carbon nanofiller (0.1–0.5–1.0–1.5–2.0 weight%—code: C) in a ball milling for 30 min at 20 Hz. The nanocomposites were identified with the code UPC with a number indicating the weight% of carbon nano-filler (code CNF). The mixing with ball mill was useful in order to induce a high degree of the filler dispersion inside the polymeric matrix, which improves both the mechanical and thermal features of the nanocomposites [5].

CNF powder was obtained by milling short carbon fibers supplied by Zoltek (Bridgeton, MO, USA) in a ball milling (mod. Retsch-MM301, 30 cycles of 10 min at 50 rpm, Retsch, Haan, Germany).

Polymeric sheets were prepared by compression molding (mod. PM 20/200, Campana S.R.L, Veduggio (MB), Italy) in a laboratory press at 200 °C for 20 min, at pressure of 20 MPa. The geometry was 60 mm × 60 mm, 2 mm thick.

More details about the material’s preparations have already been explained in detail in our previously published paper [5].

### 2.1. Materials Characterization

A pin-on-disc wear tester was used to perform wear resistance measurements in different lubricants: no lubricant (air), distilled water, artificial (simulated synovial fluid), and natural lubricant (bovine serum) [24]. Both artificial and natural lubricants were chosen in order to approximate the biological conditions of a human joint. The polymeric samples employed in this test had square shapes: 20 mm × 20 mm, and 2 mm thick. The pin was a ruby corundum grinding stones (code: M.2145 and 3 mm in diameter), at room temperature. The use of such hard material allowed us to obtain the “accelerate wear test”, in which we observed a noteworthy weight loss and improvement in the accuracy of the final result. The pin-on-disc system gives a circular shape wear trajectory with a testing load of 30 N, spin rate of 60 rpm, and test time of 120 min. Anyway, more details are given in a previous paper [4]. For each sample, the specific wear rate Ws (mm^3^/Nm) was calculated [26]:

(1) $$W_{s}=\frac{\Delta m}{𝒫xFnxL}$$

where Δm (mg) is the mass loss of the specimen, 𝒫 (g/mL) is the density (see values listed in Table 1), Fn (N) is the normal load, and L (m) is the total sliding distance. The mass loss was evaluated by a high sensitivity electronic weighing balance (mod. Explorer pro EP 214C, ASTM D1505 International standard, OHAUS Corporation, Parsippany, NJ, USA, accuracy: 10^−4^ g).

Three tracks of different diameter were evaluated after the two hours in each sample and the Ws value was the average of these three tracks. Besides, the wear test was performed in four different media: no lubricant (air), distilled water, artificial lubricant (or simulated synovial fluid), and natural lubricant (or bovine serum). The final value of the specific wear rate Ws was determined with the average of the Ws values of n.9 polymeric sheets (for each nanocomposite obtained) in the four different media. 

Data presentation and statistical analysis: the mean differences and standard deviations of specific wear rate of all the samples, with the CNF filler amount, in different media (air, distilled water, and artificial and natural lubricant), were calculated. The media (air, distilled water, and artificial and natural lubricant) and the CNF (%) nanocomposites were the independent variables. The data were first verified with the D’Agostino & Pearson test for the normality of the distribution and the Levene test for the homogeneity of variances. The data were normally distributed and homogenous; therefore, they were statistically analyzed using two-way analysis of variance (ANOVA) and Bonferroni post hoc test for multiple comparisons at a level of significance set at *p* < 0.05 (Prism 8.4.1; GraphPad Software, Inc., La Jolla, CA, USA).

Artificial lubricant was a simulated synovial fluid (SSF), formed by dissolving 0.3 wt % of hyaluronic acid in a phosphate buffered saline solution at pH = 7.4. The electrolyte concentration was as follows: Na^+^ 153.1 mM, K^+^ 4.2 mM, Cl^−^139.6 mM, phosphate buffer 9.6 mM [24].

Bovine serum, or natural lubricant, is a synovial fluid extracted from the joint of a young bovine according to current legislation. It was kept in a refrigerator at −20 °C before each test.

Density was calculated by means of the precision balance before described, equipped like a hydrostatic system that follows the Archimede’s principle. Each test was performed at room temperature for five minutes. The density (ρ) was evaluated from dry (P_dry_) and wet (P_wet_) weight of the sample, before and after the immersion in ethanol, with the following equation:

(2) $$\rho =\frac{P_{dry}}{P_{dry}-P_{wet}}{\rho_{et}}_{h}$$

where ρ_*eth*_ is equal to 0.790 g/cm^3^. The resulting density value of each sample was the average of n = 3 measurements.

### 2.2. Fourier Transform Infrared (ATR/FTIR) Spectroscopy

The chemical composition of the different materials was analyzed by attenuated total reflectance Fourier transform infrared (ATR/FTIR) spectroscopy (Shimazu, Tokyo, Japan). The spectra were obtained with the Prestige-21 FTIR spectrometer equipped with an AIM-8800 infrared microscope (Shimadzu, Tokyo, Japan), using the incorporated 3mm diameter Ge attenuated total reflectance (ATR) semicircular prism. Furthermore, the spectra were recorded with an incident angle of 30° with a resolution of 4 cm^−1^ (64 scan) and were in the range of 650–4000 cm^−1^. The Prestige software (IRsolution, version 1.10, Shimadzu, Tokyo, Japan) was used to further analyze the spectra.

### 2.3. Fourier Transform Infrared (ATR/FTIR) Spectroscopy

In order to evaluate the bioactivity, the materials were soaked in simulated body fluid (SBF) with ion concentration nearly equal to those in human blood plasma [27].

The SBF was prepared from NaCl, NaHCO_3_, KCl, MgCl_2_, 1 M HCl, CaCl_2_·6H_2_O, and Na_2_SO_4_ (Sigma-Aldrich, St. Louis, MO, USA) with a concentration that was suggested by Kokubo [27] (Table 2). The pH of the buffer was adjusted to pH 7.4 using 1 M HCl. The solution was exchanged every two days to avoid depletion of the ionic species in the SBF owing to the formation of biominerals. After 21 days of exposure at 37 °C, the samples were removed from the SBF and air-dried in a desiccator.

Fourier transform infrared (ATR/FTIR) spectroscopy (Shimadzu, Tokyo, Japan) was used to observe the characteristic peaks of hydroxyapatite layer on the surface of materials.

### 2.4. Antibacterial Activity

*Escherichia coli*, Gram-negative (ATCC 25922) and *Enterococcus faecalis*, Gram-positive (ATCC 29212) were used to evaluate the antibacterial properties of UHMWPE with 2.0 wt % of paraffin oil in function of different amounts of carbon nano-filler.

The bacterial culture was diluted in distilled water to produce a bacterial cell suspension of 10 × 10^5^ CFU/mL (where CFU is for colony forming unit). *E. coli* and *E. faecalis* were inoculated in TBX medium (Tryptone Bile X-Gluc) (Liofilchem, Italy) and in Slanetz Bartley agar base (Liofilchem, Italy), respectively.

The materials were incubated against E. *coli* for 24 h at 36 °C and against E. *faecalis* for 24 h at 44 °C. The microbial growth was evaluated by observing the diameter of the inhibition halo (ID). The obtained values are the mean standard (SD) deviation of the measurements carried out on samples analyzed three times.

### 2.5. Cytotoxicity

3-(4,5-dimethylthiazol-2-yl)-2,5-diphenyl tetrazolium bromide (MTT) assay was used to determine the metabolic activity on NIH-3T3 murine fibroblast cells. For this purpose, cells were grown in Dulbecco’s modified Eagle medium supplemented with 10% fetal bovine serum, 50.0 U/mL penicillin, and 100.0 μg/mL streptomycin, at 37 °C in a humidified atmosphere containing 5% CO_2_. When cells were seeded at a density equal to 5.0 × 10^5^ per well, onto six-well plates, they are directly exposed to disks of synthesized material. After 6, 12, and 24 h of incubation, cells were treated with MTT (500 μL; 0.50 mg/mL), previously dissolved in culture media, for 2 h at 37 °C in a 5% CO_2_ humidified atmosphere. The MTT solution was then removed and dimethyl sulphoxide (DMSO) was added to dissolve the original formazan. Finally, the absorbance at 570 nm of each well was determined using a Victor3 Perkin Elmer fluorescence and absorbance reader. The cell viability was expressed as a percentage of mitochondrial redox activity of the cells directly exposed to powders, compared with an unexposed control.

## 3. Results and Discussion

### 3.1. Wear Behavior

**Figure 1:**
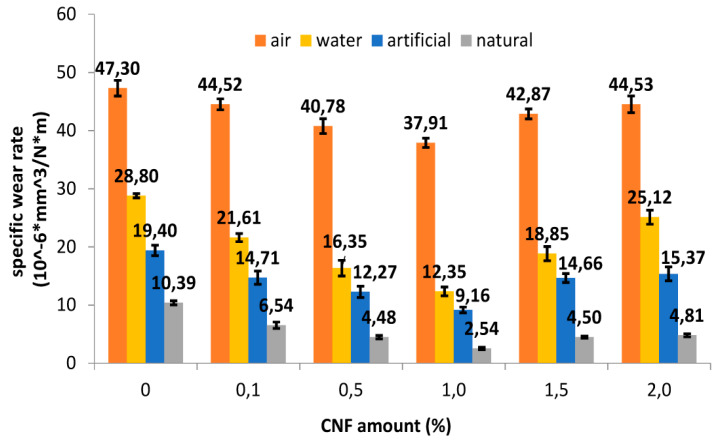
Wear rate of all of the samples vs. the carbon nano-filler (CNF) amount in different media (air, distilled water, and artificial and natural lubricant). The differences between the groups are statistically significant according to two-way analysis of variance (ANOVA) interaction and post hoc Bonferroni test, *p* < 0.0001).

Considering a comparison of the specific wear rate (Ws) of the control sample (UP) and the nanocomposites in different lubricating media reported in Figure 1, we have the following trend of Ws:

Air > Water > Artificial Lubricant > Natural Lubricant

Thus, the natural bovine serum has the best lubricant effect, while the air (or no lubricant) has the worst, as expected. The highest reduction occurs in natural lubricant, for all the samples, regardless of the CNF amount. In particular, the decrease was −78.03% in the UP control sample (from 47.3 ×·10^−6^ mm^3^/Nm to 10.39·× 10^−6^ mm^3^/Nm, *p* < 0.0001) and −89.01% (from 40.78·× 10^−6^ mm^3^/Nm to 4.48·× 10^−6^ mm^3^/Nm, *p* < 0.0001) in the UPC 0.5 wt %, and −93.29% (from 37.91 ×·10^−6^ mm^3^/Nm to 2.54 ×·10^−6^ mm^3^/Nm, *p* < 0.0001) in the UPC 1.0 wt %. These data suggest that UPC 1.0 wt % nanocomposite exhibits the highest wear rate reduction, regardless of the lubricating media. In fact, for higher filler amounts, the wear rate improves again.

This is because the filler dispersion changes with the different loads; at a low amount, such as 0.1 or 0.5 wt %, the dispersion is poor, while at higher amounts, such as 1.5 or 2.0 wt %, the great filler amount let to a filler agglomeration, which forms weak points that act as localized stress concentration points with an increases of specific wear rate improvement for an improvement of weight loss [28].The inferential analysis revealed statistically significant differences in the effect of the media (air, distilled water, and artificial and natural lubricant) on the nanocomposites (*p* < 0.0001). The statistical analysis carried out also showed that both variables (carbon nanofiller content and type of medium used) have a statistically significant effect on the wear data obtained (interaction, *p* < 0.0001).

### 3.2. Fourier Transform Infrared (ATR/FTIR) Spectroscopy

**Figure 2:**
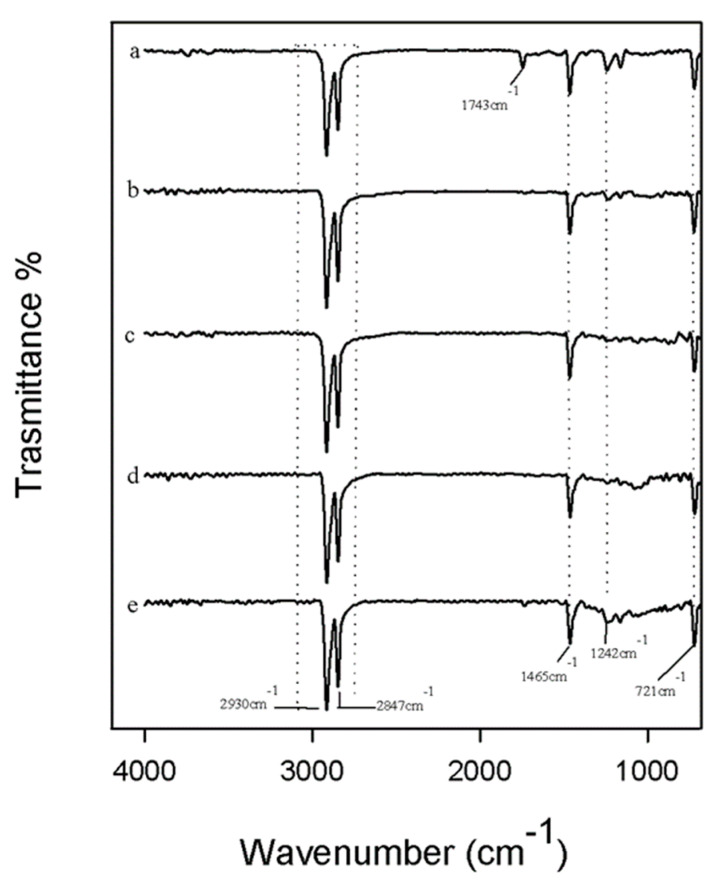
Total reflectance Fourier transform infrared (ATR/FTIR) spectroscopy spectra of (**a**) UP; (**b**) UPC 0.1 wt % CNF; (**c**) UPC 0.5 wt % CNF; (**d**) UPC 1.0 wt % CNF; and (**e**) UPC 2.0 wt % CNF. U, ultra high molecular weight polyethylene (UHMWPE); P, paraffin oil; C, carbon nanofiller.

The chemical interactions among the components in the materials and its chemical structure were evaluated using ATR/FTIR spectroscopy. In Figure 2 are reported the spectra of all samples with different percentages of carbon nano-filler (curves b, c, d, e) compared with UHMWPE with paraffin oil and without carbon nano-filler (curve a).

In all spectra, the bands at 2930 cm^−1^ and 2847cm^−1^ are observed owing to the –CH–CH asymmetric and symmetric stretching modes [29,30]. In addition, the peak at 1465 cm^−1^ was attributed to –CH asymmetrical bending, while the C–C stretching vibrations could be assigned at 1242 cm^−1^ [29,30]. Furthermore, in the spectrum of UHMWPE with paraffin oil and without carbon nano-filler (curve a), the peaks at 1743 cm^−1^ and 721 cm^−1^ were detectable. The peak at 721 cm^−1^ was reported to be related to the asymmetric angular deformation of CH_2_ groups in mineral oil [31]. The band at 1743 cm^−1^ could be attributable to an oxidative degradation process that occurs during the high temperature processing. In fact, the peak at 1743 cm^−1^ is attributed to the absorption of carbonyl species (C=O) [32], while oxygen bearing functionalities such as C–O stretching could be assigned at 1242 cm^−1^. Observing the spectra of the polymer containing the different percentage of carbon nano-filler, the peak at 1743 cm^−1^ disappeared, and it was weakly detectable in UPC 2.0 wt % CNF spectrum (Figure 2 panel E). This could be because of the ability of carbon nano-filler (CNF)to improve resistance to oxidative degradation. Indeed, the C–O stretching vibration was also more pronounced in UPC 2.0 wt % CNF spectrum. The different change in shape and absorbance of the peak at 1242 cm^−1^ could be the result of CNF C–C stretching modes overlapping [33].

### 3.3. Bioactivity Test

The bioactivity properties of the different materials were evaluated using Kokubo’s test. The materials were soaked in simulated body fluid (SBF) for 21 days, and after this exposure time, the formation of the hydroxyapatite on the surfaces of all samples was detected by ATR/FTIR.

**Figure 3:**
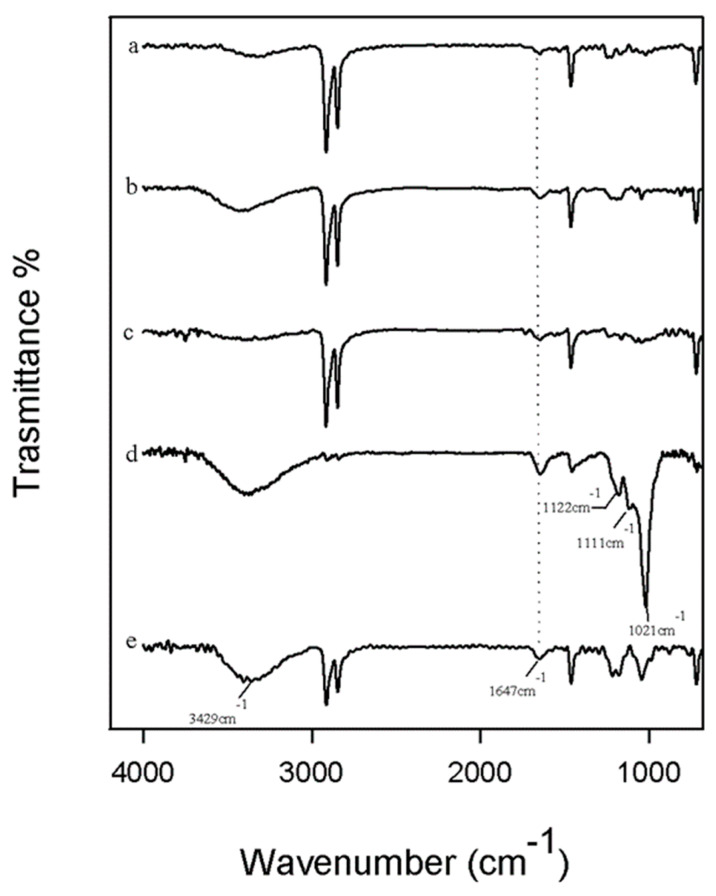
Total reflectance Fourier transform infrared (ATR/FTIR) spectroscopy spectra of (**a**) UP; (**b**) UPC 0.1 wt % CNF; (**c**) UPC 0.5 wt % CNF; (**d**) UPC 1.0 wt % CNF; and (**e**) UPC 2.0 wt % CNF after 21 days in simulated body fluid (SBF).

In Figure 3, the spectra of all materials are reported, in which it is possible to observe the peaks at 3429 and 1647 cm^−1^ that correspond to the −OH groups [34,35]. Comparing all materials, in the spectra of UHMWPE with paraffin oil and with 1.0 wt % of carbon nano-filler (curve d), the typical bands of the hydroxyapatite layer are clearly visible. In fact, the peak at 1021 cm^−1^ is the result of the absorption of the stretching mode of the PO_4_^−3^ ions [36,37,38]. In addition, the P−O bond of the phosphate group appears at 1122 and 1111 cm^−1^ [39]. Furthermore, only in this spectrum, the typical peaks of the polymer are not very intense, which is probably because the material’s surface is covered by a hydroxyapatite layer that is very thick compared with the others [40,41]. In conclusion, all materials seem to be bioactive because the hydroxyapatite bands are present in all spectra, but when carbon nano-filler is added to 1.0 wt %, the hydroxyapatite peaks are more intense, suggesting that this material is the most bioactive of all.

According to the wear resistance test result, the optimal wear performance and optimal bioactivity are noticed in the same nanocomposite, containing 1.0 wt % of carbon nano-filler. This result confirms that the filler dispersion is a key parameter to achieve the best mechanical as well as biological response, as discussed in Section 3.1.

### 3.4. Antibacterial Activity

**Figure 4:**
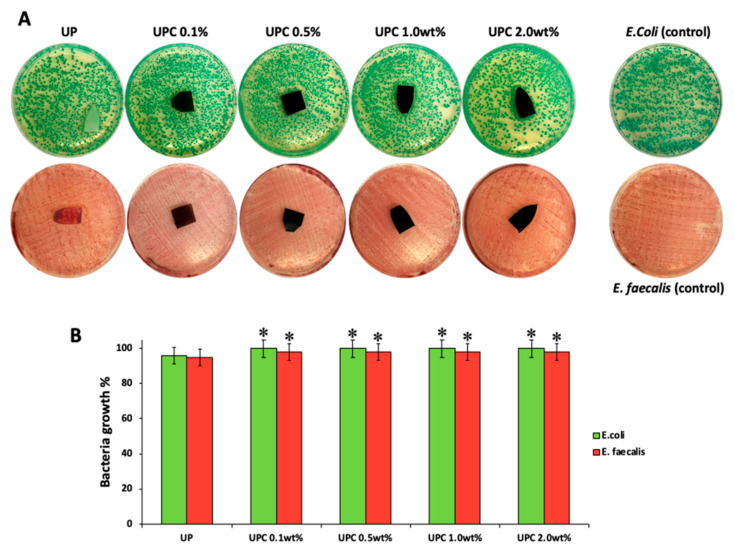
(**A**) Representative image of E. coli and E. faecalis incubated with all materials. (**B**) Bacteria growth (%) of E. coli and E. faecalis incubated with UP, UPC 0.1 wt %, UPC 0.5 wt %, UPC 1.0 wt %, and UPC 2.0 wt %. Values are the mean SD of measurements carried out on samples analyzed three times. The means and S.D. are shown. * *p* < 0.05 vs. the bacteria control treated without materials.

The materials with the same percentage of paraffin oil and several amounts of carbon nano-filler were incubated with gram-positive and gram-negative bacterial strains. In Figure 4A, the representative images of both bacteria are reported, while the bacterial growth is shown in Figure 4B. The UP material with only 2.0 wt % of paraffin oil was used as control in order to evaluate the effects of carbon nano-filler on bacterial growth. In all plates, after 24 h of incubation against E. coli and E. faecalis, the inhibition halo (ID) was not observed, regardless of the carbon nano-filler content. Therefore, the results show that the materials do not have toxic effects against gram-positive and gram-negative bacterial strains. In addition, the materials during the incubation period did not release any product that could damage the bacteria growth, suggesting that they could be used as optimal materials for joint replacement (JR) in artificial knees and hips prosthesis.

### 3.5. Cytotoxicity

**Figure 5:**
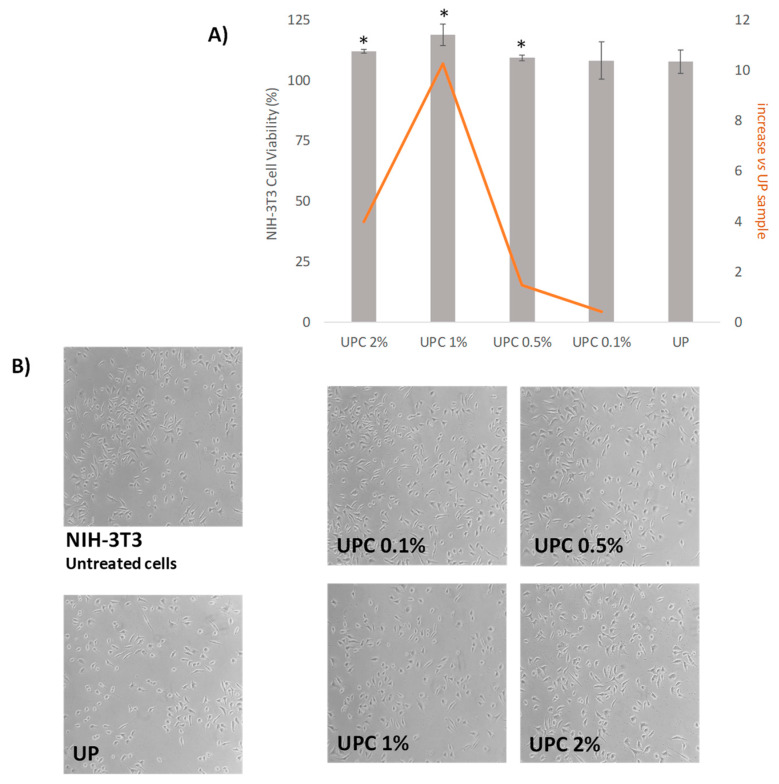
(**A**) Data from MTT assay expressed as NIH-3T3 cells viability (%) vs. untreated cells. Values are the mean ± SD of two independent experiments performed in triplicate. *p < 0.05 vs. untreated cells. The increase in cell viability vs. UP sample is also shown. (**B**) Representative cell morphology images of NIH-3T3 cells after treatment with investigated samples. Images were acquired by Inverted Phase Contrast Brightfield Zeiss Primo Vert Microscope.

The nanocomposites were investigated for their biocompatibility through MTT-direct contact test. The data (Figure 5) acquired highlighted that all the samples avoided cytotoxic effects, and that the increase in amount percentage in carbon nano-filler positively affected NIH-3T3 viability. In fact, all the samples appeared to induce a cell growth increase with respect to UP material, reaching its maximum by the UPC1% sample. This latter showed a percentage increase in cell viability equal to 10.2% compared with UP. Thus, the incorporation of CNFs in a small dose level percentage seemed to not exert evident redox mitochondrial modification. Previous studies evidenced that multiwall carbon nanotubes caused a significant time- and dose-dependent decrease in cells’ viability [42,43], and that they could be toxic at sufficiently high concentrations. Indeed, it was reported that carbon nanomaterials present significantly different cytotoxicity depending on their physicochemical properties, including size, length, shape, and surface area [44,45,46]. The lack of filler coalescence, defined as a consequence of CNF incorporation, suggested the constitution of an improved UHMWPE-based material, in which the absence of debris could favour the slowing down of inflammatory and oxidative stress effects commonly ascribed to these materials [47,48,49,50,51,52].

## 4. Conclusions

In this paper, we investigated the biological and antibacterial activity of wear resistant nanocomposites based on biomedical grade UHMWPE paraffin oil and carbon nano-filler. Paraffin oil favors the workability of the typical high viscosity UHMWPE and the filler dispersion within the polymer. Carbon nanofiller act as lubricant to lower the friction between the UHMWPE and other counterparts. Furthermore, carbon nano-filler prevents the oxidative degradation of the nanocomposite during its processing, as observed by the FTIR analysis.

The wear test was performed in different lubricating media (no lubricant or air, distilled water, simulated synovial fluid, and bovine serum). The lubricating effect was in the following order: natural lubricant > artificial lubricant > water > air. The more wear resistant nanocomposite resulted it being reinforced with 1.0 wt % of CNF regardless of the lubricating media because of the best filler dispersion within the polymeric matrix. A filler load lower than 1.0 weight percentage, as well as a higher one, are too little and too much, respectively, in order to achieve a homogeneous dispersion and to avoid filler coalescence, respectively. This aspect also reflects the bio-activity response of the materials, which has the best hydroxyapatite production in just the 1.0 wt % sample.

This suggests that it could be applicable in artificial prosthesis. In order to verify in more depth the suitability of these nanocomposites, further studies are underway on the release of components over time into biological fluids.

## Figures and Tables

**Table 1:** Values of reference UP and of the nanocomposites at different immersion times. U, ultra high molecular weight polyethylene (UHMWPE); P, paraffin oil; C, carbon nanofiller.

Sample Code	Density (g/mL)
UP	0.866 ± 0.001
UPC 0.1 wt %	0.864 ± 0.001
UPC 0.5 wt %	0.862 ± 0.002
UPC 1.0 wt %	0.862 ± 0.004
UPC 1.5 wt %	0.863 ± 0.002
UPC 2.0 wt %	0.865 ± 0.002

**Table 2:** Body fluid (simulated body fluid, SBF) composition.

Ion	Concentration/mol m^3^	
	SBF	Human Blood Plasma
**Na^+^**	142.0	142.0
**K^+^**	5.0	5.0
**Mg^2+^**	1.5	1.5
**Ca^2+^**	2.5	2.5
**Cl^−^**	147.8	103.0
**HCO_3_^−^**	4.2	27.0
**HPO_4_^2−^**	1.0	1.0
**SO_4_^2−^**	0.5	0.5
